# Continuous Drip Flow System to Develop Biofilm of *E. faecalis* under Anaerobic Conditions

**DOI:** 10.1155/2014/706189

**Published:** 2014-10-13

**Authors:** Ana Maria Gonzalez, Erika Corpus, Amaury Pozos-Guillen, Daniel Silva-Herzog, Antonio Aragon-Piña, Nestor Cohenca

**Affiliations:** ^1^Endodontics Postgraduate Program, Faculty of Dentistry, The Autonomous University of San Luis Potosí, Manuel Nava 2, 78290 San Luis Potosí, SLP, Mexico; ^2^Basic Sciences Laboratory, Faculty of Dentistry, The Autonomous University of San Luis Potosí, Manuel Nava 2, 78290 San Luis Potosí, SLP, Mexico; ^3^Faculty of Engineering, The Autonomous University of San Luis Potosí, Manuel Nava 8, 78290 San Luis Potosí, SLP, Mexico; ^4^Department of Endodontics and Department of Pediatric Dentistry, School of Dentistry, University of Washington, 6222 NE 74th Street, Seattle, 98115 WA, USA

## Abstract

*Purpose.* To evaluate a structurally mature *E. faecalis* biofilm developed under anaerobic/dynamic conditions in an *in vitro* system. *Methods.* An experimental device was developed using a continuous drip flow system designed to develop biofilm under anaerobic conditions. The inoculum was replaced every 24 hours with a fresh growth medium for up to 10 days to feed the system. Gram staining was done every 24 hours to control the microorganism purity. Biofilms developed under the system were evaluated under the scanning electron microscope (SEM). *Results.* SEM micrographs demonstrated mushroom-shaped structures, corresponding to a mature *E. faecalis* biofilm. In the mature biofilm bacterial cells are totally encased in a polymeric extracellular matrix. *Conclusions.* The proposed *in vitro* system model provides an additional useful tool to study the biofilm concept in endodontic microbiology, allowing for a better understanding of persistent root canal infections.

## 1. Introduction

Contemporary microbiology has demonstrated that microorganisms are organized under specific environmental conditions [1]. Planktonic microorganisms are single cells that may float or swim in a liquid medium. In contrast, under specific environmental conditions, phenotypic adaptations are expressed as a form of life termed biofilm. Biofilm is characterized by the immobilization on a surface, cell-cell interactions, formation of microcolonies, excretion of extracellular polymers (EPS), and development of three-dimensional structures which confer protection in order to ensure their permanence [1, 2].

Mature biofilm is a complex heterogeneous structure of dormant and actively growing bacteria colonies along with enzymes, excretory products, and small channels forming part of the overall structure [3]. It is also known that a mature biofilm is able to tolerate antimicrobial concentrations of 10 to 1000 times that required to remove planktonic bacteria [4]. The biofilm concept has changed and it is currently defined as “a bacterial community immersed in a liquid medium, characterized by one or more bacteria that are attached to each other, to a substrate or surface and embedded in an extracellular matrix produced by them, and shows an altered phenotype in the degree of cell proliferation or the expression of their genes” [5]. Thus, the study of biofilms has skyrocketed in recent years due to the increased awareness of the pervasiveness and impact of biofilms on natural and industrial systems, as well as human health (chronic bacterial prostatitis, cyst fibrosis, and periodontitis) [5].

Oral biofilms are unique mainly due to the fact that they may form under highly different factors. In the oral cavity, biofilms are found as dental plaque over the enamel surface, over the root surface in periodontal disease, and in the internal and external surface of roots suffering pulpal and periapical pathology. In the category of secondary endodontic infections,* E. faecalis* is the microorganism most often associated with asymptomatic chronic periradicular lesions, while not being as prominent in acute periapical periodontitis or acute periradicular abscess [6–9].

It is reported to be present in 4 to 40% of secondary endodontic infections. The frequency of* E. faecalis* in persistent lesions has been shown to be much higher. Previous work related to refractory periapical lesions has shown a prevalence ranging from 24 to 77%. In some cases it has been found as the only inhabitant in teeth endodontically treated [6, 10, 11]. Current literature has demonstrated that biofilms may remain viable in anatomical areas of the root canal system that remain untouched by either mechanical or chemical disinfection [12–14]. Clinical examination of root tips associated with refractory periapical periodontitis has suggested the presence of bacterial biofilm at the apical portion of the root canal [15–18].* In vitro* studies have focused on the efficacy of selected irrigants and medicaments to arrest biofilms grown in different substrates [19–23], using strains of selected species and nonputative strains from the root canal. However, there is a lack of information relating to biofilm formation capabilities and characteristics of clinical isolates recovered from the root canals. Moreover, the development and validation of practical, reproducible, and clinically relevant laboratory models for the study of biofilms are still challenging. The aim of the present study was to evaluate a structurally mature* E. faecalis* biofilm developed under anaerobic/dynamic conditions in an* in vitro* system.

## 2. Material and Methods

**Figure 1:**
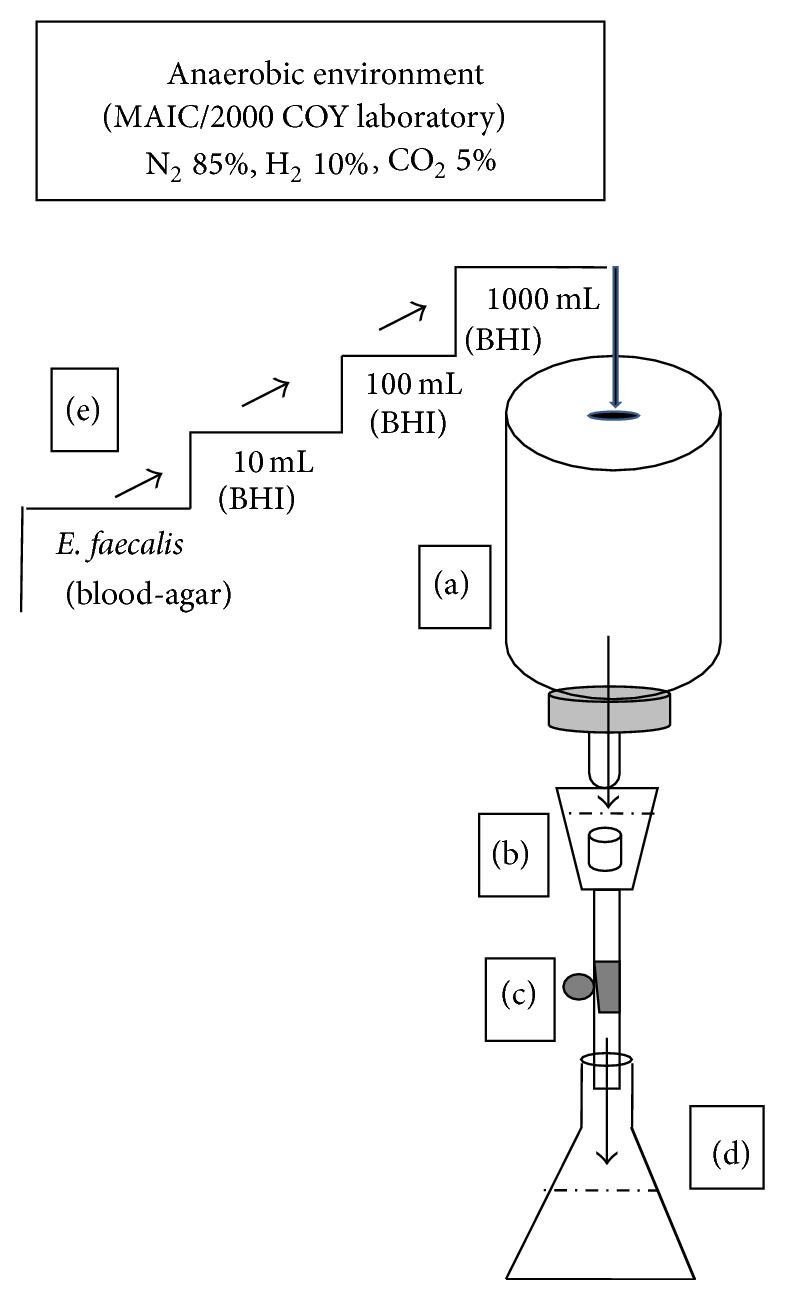
Experimental device developed under a continuous drip flow system and designed for dynamic biofilm formation under anaerobic conditions. Plastic container with fresh growth medium (a) connected to a plastic chamber containing the specimen (b). The flow was controlled to a velocity of 0.5 mL/min (c) and the waste collected on a second container (d). Stepwise scale-up strategy to reach a final concentration of 1.5 × 10^8^ cells/mL (e).

An experimental device was created using a continuous drip flow system designed to develop biofilm under anaerobic conditions (Coy Laboratory Products, Grass Lake, MI, USA) (Figure 1). This experimental device was previously sterilized and built as follows. A plastic container (Figure 1(a)) with fresh growth medium was connected by tubing to a plastic chamber containing the specimen (Figure 1(b)) on which the biofilms were formed. Twenty specimens were performed as follows. The apical 3 mm of teeth recently instrumented and extracted was removed from each root. Samples were taken to an ultrasonic bath (BioSonic UC50, Coltene/Whaledent Inc., NJ, USA) soaking in EDTA (Fermont, Monterrey Chemicals, SA, Mexico) to 17% for 4 min and then samples were immersed in 5.25% sodium hypochlorite for 4 min to remove organic and inorganic tissue. The specimens were then sterilized at 121°C and 15 pounds of pressure for 20 min. A second container (Figure 1(d)) for waste collection was used. The flow was controlled to a velocity of 0.5 mL/min (Figure 1(c)).

The inoculum of* E. faecalis* previously developed and identified in root tips associated with endodontic failure was replaced every 24 hours with a fresh growth medium for up to 10 days to feed the system. The fresh medium was brain-heart infusion broth (BBL Becton Dickinson Mexico, Cuautitlan, Mexico). The inoculums were prepared using a stepwise scale-up strategy in a proportion of 1 : 10 (Figure 1(e)) to reach a final concentration of 1.5 × 10^8^ cells/mL, adjusted to 0.5 Mac Farland turbidity standard. This step is repeated from 100 to 1000 mL to obtain the volume required by the experimental device in order to be replaced in the continuous flow system every 24 hours for the time necessary for the experimental phase. Gram staining was done every 24 hours to control the microorganism purity.

All the samples were prepared for SEM analysis as follows: they were gently washed with 0.1 M phosphate buffered solution, fixed with 2% glutaraldehyde (Sigma-Aldrich, St. Louis, MO, USA) and 1% Alcian Blue Stain 8GX (Sigma-Aldrich, St. Louis, MO, USA), and stored at 4°C for 24 hours. Once fixed, samples were washed three times with 0.1 M phosphate buffer solution to remove excess material. The samples were dehydrated in a series of anhydrous ethanol (Industrial Chemical Technology, Ltd.) 20%, 40%, 60%, 80%, 90%, and 95% for 10 minutes in each series to be left finally immersed in ethanol 100% and critical point dried in a critical point dryer (CPD 030 BAL-TEC GmbH, Schalksmühle, Germany). The samples were sputter coated with 20 nm gold-palladium mixture (Fine Coat Ion Sputter JFC-1100, USA). Biofilms specimens were evaluated under the SEM (JEOL JSM-6610 LV, JAPAN) at a 5 Kv accelerating potential at different magnifications.

## 3. Results

**Figure 2:**
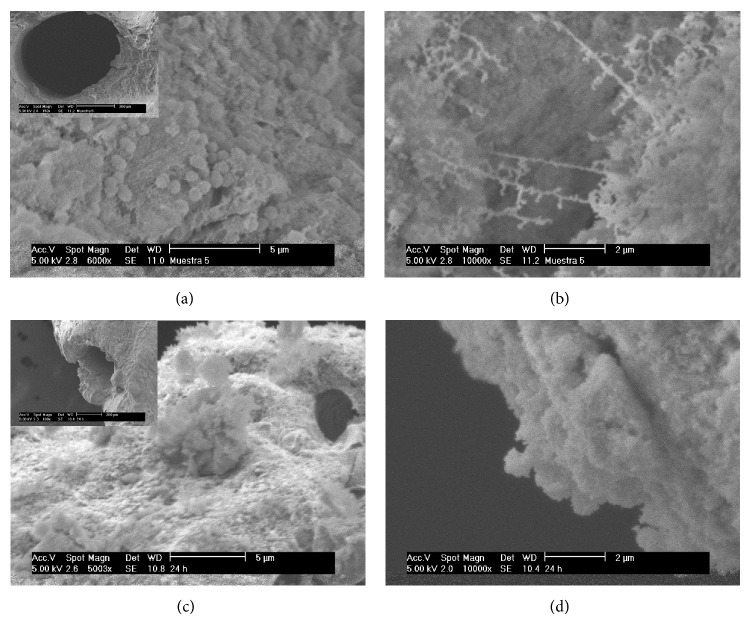
*In vitro E. faecalis* biofilm formed in an anaerobic/dynamic system. ((a) and (c)) Coccoid structures attached to mature biofilm (6000x). (b) Extrapolymeric fibers (10000x). (d) “Mushroom-shaped structures” (10000x).

SEM analysis revealed bacterial biofilm in all samples. Careful observation of these structures under higher magnification revealed clumps of coaggregated bacterial cells in a matrix of extracellular polymeric substance.* E. faecalis* biofilms displayed a complex three-dimensional structure which demonstrated spatial heterogeneity and a typical architecture showing microcolonies with ramifying water channels. Fibrillar structures appeared to be made up of twisted fibers. Larger structures of wrapped sheets were also present and consisted of small numbers of bacteria cells embedded in a matrix of fibers. The* E. faecalis* biofilm, grown under anaerobic conditions, showed a clump of bacteria cells attached to the dentine surface (Figure 2(a)). The fibers were more apparent and formed irregular, net-like structures (Figure 2(b)). Coaggregated bacterial cells in a polymeric matrix were observed forming “mushroom-shaped structures,” corresponding to a mature biofilm with cells totally encased in a polymeric extracellular matrix (Figures 2(c) and 2(d)).

## 4. Discussion

The system discussed in this study is based on a modified Robbins device (MRD) developed by Jim Robbins to allow the reproducible and simultaneous formation of biofilms exposed to a fluid flow [24–26]. MRD is filled with a suspension of microorganisms and is inverted to improve the adhesion of the planktonic cells to the discs. Based on that model, an experimental device was developed using a disposable infusion pump in which it was possible to maintain a constant flow of culture medium infected with the microorganism on dentin surfaces for 10 days, time required to carry out the process of redistribution of cells adhered by mobility on the surface, cell division and aggregation, and finally the excretion of polymers with the consequent formation of a mature biofilm [27–29].

Based on our results and in concordance with Dunavant et al. [19] continuous flow of nutrients is the key to developing a mature and robust biofilm. As demonstrated in the present study, we successfully created an* in vitro* model system capable of developing* E. faecalis* biofilms under anaerobic conditions using a continuous flow of nutrients. The flow distribution is an important factor, allowing bacteria to make secondary colonization and contribute to developing a more robust biofilm in areas with minor nutrients. Thein et al. [30] have found that, in comparison with static conditions, dynamic conditions have a significant positive impact on microbial biofilm growth. This novel research model simulates clinical conditions in which disintegration of the sealer or undetected voids in the filling mass may create leakage channels that allowed periradicular tissue fluids to reach residual bacteria within tubules and provide nutrient for their growth [31].

The results of the present study also demonstrate that* E. faecalis* is capable of forming biofilms under anaerobic conditions. To our knowledge, this is one of the few studies which evaluate* E. faecalis* biofilm formation under anaerobic conditions. The study is based on the fact that root canal conditions and moreover the periapical area are under strict anaerobic environment. The biofilm mode of growth is a survival strategy and harsh environmental conditions existing in the root canal may favor the growth of bacteria as a biofilm. George et al. [32] found that when* E. faecalis* was grown under anaerobic and nutrient rich conditions, a matured biofilm with apparent water channels on the root canal was observed. On the other hand, when cultured aerobically the typical biofilm structure with mushroom-shaped microcolonies was not observed.

The current microstructural findings show that a mature biofilm formation was achieved by the end of 10 days. SEM images show an irregular layer on the dentin which consists of ovoid (similar to bacilli), elongated, and filamentous forms. Also, some coccoid forms wrapped in a kind of sheath which joined others forming an elongated structure, similar to those described for the first time by Ramachandran Nair [33]. Water channels as well as mushroom-shaped microcolonies were observed. This study suggests that hydrodynamics and environmental conditions are a significant factor in the quality of* E. faecalis* biofilm development. It is important to mention that the literature has described numerous systems of biofilm formation, both static and dynamic, and their applications in endodontics using* E. faecalis* as a microorganism of study due to its resistance to endodontic treatment [34]. However, a system in anaerobic conditions is difficult to realize. The model proposal in this study has the advantage of being easy to assemble in an anaerobic chamber; it can be to perform multiple systems at once; there is less risk of contamination because the system is not reusable; it is possible to change the flow if required; it is possible to control the time from hours to days to observe and study the formation of the biofilm thickness. It is also possible to obtain sufficient quantities of biofilm in order to realize different assays. Further studies with nondestructive chemical analytical techniques are necessary to increase our knowledge of the biofilm chemical composition in this model system.

## 5. Conclusion

The proposed* in vitro* system model provides an additional useful tool to study the biofilm concept in endodontic microbiology, allowing for a better understanding of persistent root canal infections.
